# Cost-Effectiveness Analysis of Four Simulated Colorectal Cancer Screening Interventions, North Carolina

**DOI:** 10.5888/pcd14.160158

**Published:** 2017-02-23

**Authors:** Kristen Hassmiller Lich, David A. Cornejo, Maria E. Mayorga, Michael Pignone, Florence K.L. Tangka, Lisa C. Richardson, Tzy-Mey Kuo, Anne-Marie Meyer, Ingrid J. Hall, Judith Lee Smith, Todd A. Durham, Steven A. Chall, Trisha M. Crutchfield, Stephanie B. Wheeler

**Affiliations:** 1 Department of Health Policy and Management, University of North Carolina at Chapel Hill, Chapel Hill, North Carolina; 2 Department of Industrial and Systems Engineering, North Carolina State University, Raleigh, North Carolina; 3 Lineberger Comprehensive Cancer Center, University of North Carolina at Chapel Hill, Chapel Hill, North Carolina; 4 Center for Health Promotion and Disease Prevention, University of North Carolina at Chapel Hill, Chapel Hill, North Carolina; 5 Division of General Medicine and Clinical Epidemiology, University of North Carolina at Chapel Hill, Chapel Hill, North Carolina; 6 Sheps Center for Health Services Research, University of North Carolina at Chapel Hill, Chapel Hill, North Carolina; 7 Division of Cancer Prevention and Control, Centers for Disease Control and Prevention, Atlanta, Georgia; 8 Department of Epidemiology, University of North Carolina at Chapel Hill, Chapel Hill North Carolina; 9 Renaissance Computing Institute, University of North Carolina at Chapel Hill, Chapel Hill, North Carolina

## Abstract

**Introduction:**

Colorectal cancer (CRC) screening rates are suboptimal, particularly among the uninsured and the under-insured and among rural and African American populations. Little guidance is available for state-level decision makers to use to prioritize investment in evidence-based interventions to improve their population’s health. The objective of this study was to demonstrate use of a simulation model that incorporates synthetic census data and claims-based statistical models to project screening behavior in North Carolina.

**Methods:**

We used individual-based modeling to simulate and compare intervention costs and results under 4 evidence-based and stakeholder-informed intervention scenarios for a 10-year intervention window, from January 1, 2014, through December 31, 2023. We compared the proportion of people living in North Carolina who were aged 50 to 75 years at some point during the window (that is, age-eligible for screening) who were up to date with CRC screening recommendations across intervention scenarios, both overall and among groups with documented disparities in receipt of screening.

**Results:**

We estimated that the costs of the 4 intervention scenarios considered would range from $1.6 million to $3.75 million. Our model showed that mailed reminders for Medicaid enrollees, mass media campaigns targeting African Americans, and colonoscopy vouchers for the uninsured reduced disparities in receipt of screening by 2023, but produced only small increases in overall screening rates (0.2–0.5 percentage-point increases in the percentage of age-eligible adults who were up to date with CRC screening recommendations). Increased screenings ranged from 41,709 additional life-years up to date with screening for the voucher intervention to 145,821 for the mass media intervention. Reminders mailed to Medicaid enrollees and the mass media campaign for African Americans were the most cost-effective interventions, with costs per additional life-year up to date with screening of $25 or less. The intervention expanding the number of endoscopy facilities cost more than the other 3 interventions and was less effective in increasing CRC screening.

**Conclusion:**

Cost-effective CRC screening interventions targeting observed disparities are available, but substantial investment (more than $3.75 million) and additional approaches beyond those considered here are required to realize greater increases population-wide.

## Introduction

Colorectal cancer (CRC) is the third leading cause of cancer deaths in the United States. Nearly 140,000 people are diagnosed with the disease each year and more than 50,000 die from the disease (1). Screening can detect CRC at a localized stage when treatment is most effective and can detect and remove precancerous polyps, thereby reducing incidence and death (2,3). National guidelines recommend routine CRC screening for average-risk adults aged 50 through 75 years (2). However, a national survey based on self-report from 2010 suggests that only 64.5% (4) of age-eligible people meet these guidelines. These self-reported data probably overestimated actual screening (5). In addition, screening rates were lower among the uninsured compared to the insured and among people with low incomes or low educational levels compared to their higher income and education counterparts (4,6). Because of the large differences in screening rates and corresponding disease outcomes across these subpopulations (4), addressing disparities in receipt of screening is essential.

Tested interventions have increased screening in populations with observed disparities in receipt of CRC screening. For example, one multifaceted intervention supported screening among low-income patients in community health centers through mailed information, screening reminders, and outreach by patient navigators (7). However, such interventions have not been implemented on a wide scale and have not been compared with alternatives to determine their relative cost and effectiveness and taken together, their efficiency. Decision makers need this information for interventions to inform their recommendations, policies, and decisions about investment in CRC screening programs. Because such decisions are often made at the state level, we chose to evaluate CRC interventions in a single state, North Carolina. The factors that shape screening preferences, access, and, ultimately, receipt and disparities in screening suggest that intervention programs should be tailored to current levels of health care, population characteristics, and access to care in a given geographic context or when targeting a specific subpopulation. Our objective was to compare the impact and cost-effectiveness of 4 evidence-based interventions for increasing CRC screening and reducing disparities in guideline-concordant CRC screening in North Carolina and to present a process that could be replicated to inform decision making about CRC interventions in other states.

## Methods

We used an individual-based simulation model to estimate the relative effects of 4 evidence-based approaches to increasing CRC screening among age-eligible (aged 50 to 75 years at some point during the intervention window of January 1, 2014, and December 31, 2023) North Carolina residents in whom disparities in guideline-concordant receipt of screening were observed — most notably among subgroups by sex, race, insurance status, and county of residence (6). Individual-based modeling is computer modeling in which events are simulated, with realistic uncertainty built in, for each heterogeneous individual in a specified population based on predefined rules (eg, incidence rates, mortality rates, interaction between individuals or with the environment) over a specified period to estimate population-level outcomes over time or the impact of simulated interventions. We reviewed the literature and US Preventive Services Task Force guidelines (2) to develop and refine 4 intervention scenarios through a series of interviews with 19 decision makers and other local stakeholders (ie, clinicians, public health professionals, payers, researchers, state health officials, and experts from the Centers for Disease Control and Prevention). Although the same intervention scenarios we selected may not be the ones preferred in other settings, they were among the most common approaches documented in the literature and were endorsed by our stakeholders. These interventions consisted of mailed reminders to a registry of Medicaid enrollees, expansion of endoscopy facilities to increase overall access to care, a mass media campaign targeting African Americans, and screening colonoscopy vouchers for the uninsured.

We used a model that is an extension of a validated CRC screening model based on the MISCAN-COLON model (8,9). Our primary change to the model was to simulate a complete, realistic population of all North Carolina residents who were age-eligible (aged 50–75 y) for routine CRC screening at some point during the 10-year intervention period, and to project each individual’s screening method and compliance with recommended CRC screening on the basis of multilevel, multivariable statistical models, estimated by using rich North Carolina public and private claims data. The individual-based model effectively projects known screening patterns to the full population, first in this claims-based “screening-as-usual” scenario representing currently observed status-quo patterns of CRC screening receipt. This model provided a virtual world in which we implemented the 4 intervention scenarios, each targeting synthetic individuals with different characteristics, to learn about their relative cost, impact on CRC screening receipt, and efficiency — defined as cost per additional life-year up to date with screening recommendations. This study was approved by the University of North Carolina Chapel Hill institutional review board.

### Model overview and outcomes

The model simulated the full life course of all North Carolina residents who were age-eligible for CRC screening at some point during our study’s 10-year intervention window of January 1, 2014, through December 31, 2023. We simulated a “screening-as-usual” scenario (existing screening practices) and compared its CRC screening outcomes to those under hypothetical intervention scenarios. The model simulated a person’s status, over time, as up to date with testing, defined as having had a fecal occult blood test (FOBT) (most observed fecal tests were FOBTs) within the past year or a colonoscopy within the past 10 years. To model actual screening at the beginning of the intervention window, simulated screening began when all people included in the synthetic population were age-eligible – even if this occurred prior to January 1, 2014. Increases in screening resulting from the interventions began January 1, 2014, and ended December 31, 2023, ensuring the full impact of any screening colonoscopies received in the last year of the intervention window were fully included in intervention effects.

Primary outcomes were the percentage of age-eligible people who were up to date with recommended CRC screening after 10 years of intervention (a point-in-time measure) and the number of additional life-years synthetic individuals were up to date with CRC screening over the entire study period under each intervention scenario (4 interventions simulated independently as well as combination scenarios) compared with the screening-as-usual scenario; the total cost of each intervention (independently simulated); and the incremental cost per additional life-year synthetic individuals were up to date with recommended CRC screening because of the intervention. Secondary outcomes were reduction in disparities in the percentage of age-eligible people up to date with CRC screening recommendations, by sex, race, insurance type, and geographic location.

### Simulated population

We sought to simulate routine screening on the basis of real-world (observed) combinations of known determinants of CRC screening: age, sex, race, insurance type, and geographic location (6). A primary input to the model was a synthetic population of North Carolina residents, which was created using data from the American Community Survey Public Use Microdata Sample, 2005–2010 (www.census.gov/acs/www). A total of 3,918,469 simulated people were age-eligible for screening sometime during the intervention window. Table 1 presents a snapshot of the population on the first day of the intervention window. We assigned each person in the synthetic population a life expectancy based on their observed age, race, and sex, according to life tables (10). Insurance coverage was absent from the synthetic population and was assigned probabilistically.

**Table 1:** Demographic Characteristics of the Simulated Population of North Carolina Residents Age-Eligible^a^ for Colorectal Cancer Screening on January 1, 2014^b^

Characteristic	Overall	Aged 50–64 y^c^	Aged 65–75 y^c^
**Population, n**	2,782,559	1,844,279	938,280
**Sex**			
Male	47.7	48.0	47.1
Female	52.3	52.0	52.9
**Race**			
White	77.0	75.3	80.3
African American	18.4	19.5	16.4
Other^d^	4.6	5.2	3.3
**Insurance**			
Uninsured	10.3	15.5	0.1
Private	49.2	73.9	0.4
Medicare only	31.6	3.5	86.9
Medicaid only	3.1	4.7	0.0
Medicare and Medicaid	5.8	2.4	12.6

a Aged 50 to 75 years.

b Values are percentages unless otherwise indicated..

c Age on January 1, 2014.

d Asian/Pacific Islanders, American Indian/Alaska Natives, and other racial minorities. Hispanic ethnicity is distributed across racial groups.

### Receipt of CRC screening

We calculated predicted probabilities for receipt of any CRC screening test and the type of screening method (colonoscopy vs FOBT) for synthetic individuals on the basis of their demographic characteristics by using 2 sets of nested insurance-claims–derived logistic regression models (6). Models of both screening test use and type of test were multilevel, with county-level (eg, number of primary care providers per 10,000 residents) and individual-level attributes (eg, insurance type, race, sex) predicting whether or not a CRC screening test was received, and, if so, what method. Method was limited to colonoscopy or FOBT because of the very small proportion of people in North Carolina who received other types of CRC screening. The analytical sample used to estimate these statistical models included all North Carolina residents turning 50 between January 1, 2003, and December 31, 2008, who were continuously enrolled in private health plans, Medicaid, or Medicare during this 6-year period. Predicted probabilities estimated from these statistical models were scaled up proportionally for all individuals to adjust claims-based estimates of overall screening on the basis of national survey data, adjusted for self-report bias (5).

### Diagnostic testing and surveillance

Diagnostic testing and surveillance, although not the focus of this study, were simulated with the objective of estimating up-to-datedness attributable to positive results from routine screenings and follow-up diagnostic colonoscopies triggered by simulated interventions. As such, people receiving FOBTs who had positive test results were presumed to have been offered diagnostic colonoscopies, and 75% were presumed to receive follow-up colonoscopy (8). Following the detection and removal of precancerous polyps, they entered a period of surveillance during which they were offered colonoscopies every 3 years, and adherence was presumed to be 80% (8). After 2 negative colonoscopies, determined probabilistically, these people transitioned from surveillance back to their original schedule of routine screening.

### Analysis

The model was implemented in AnyLogic, version 6.9 (Product Marketing Corp). Model testing consisted of independent code review, extreme-value testing, behavior-reproduction testing in which simulated trends were compared with available data, and review of model behavior with context experts (11). To ensure that differences in outcomes were due to interventions and not randomness, innovative “common patients” methods developed in engineering were implemented to ensure common random numbers were used for all variables unrelated to intervention scenarios across the screening-as-usual and intervention scenarios (12).

Each intervention was implemented individually and in combinations. Combination intervention scenarios added the 4 interventions, one at a time, based on their impact on life-years up to date with screening, from greatest to least impact. Incremental cost-effectiveness was defined as the ratio of the difference in cost between the intervention scenario and screening-as-usual scenario, divided by the difference in life-years up to date between the intervention scenario and screening as usual.

For the secondary analysis, the impact of each intervention scenario in sub-populations was estimated to quantify the ability of each intervention to address screening disparities in the intervention compared with screening-as-usual scenarios 10 years after the interventions were implemented. Given that simulated cohorts were extremely large, 10 replications were run to assess variability in outcomes; mean results were reported.

### Intervention scenarios

Four intervention approaches were developed and quantified based on systematic literature review and extensive stakeholder interviews and were designed to reflect a balance of approaches under consideration in North Carolina with evidence about how to address observed CRC screening disparities. The costs of each intervention were estimated from the state’s perspective and reflected the expected resources needed for implementation of each intervention scenario above and beyond what would be covered under usual care (for example, the cost of the endoscopy expansion program included the cost of increasing the number of screening facilities, but not the cost of additional CRC screening that would result from increased access and would be paid under existing mechanisms). With state-supported intervention programs in mind, all 4 interventions were designed to cost less than a maximum budget of $4 million over 10 years. Detailed cost estimates are presented in Table 2.

**Table 2:** Cost Parameters for Intervention Scenarios, Cost-Effectiveness Analysis of Four Simulated Colorectal Cancer Screening Interventions, North Carolina, January 1, 2014–December 31, 2023

Cost Component	Cost Estimate, $	Notes and Sources
**Mailing reminder to Medicaid enrollees**		
Develop registry and reminder content (one-time)	10,000	Programmer and physicians’ time (author assumption)
Programming time to identify enrollees	200/y	Author assumption
Materials	0.71/reminder	Postage, paper, ink^a^
Staff time to prepare and mail reminders	3,850/y	200 h staff time
**Expansion of the number of endoscopy facilities**		
Financial incentive to locate 6 facilities in underserved areas	500,000/facility	Author assumption based on North Carolina stakeholder interviews
**Mass media campaign targeting African Americans**		
Purchase advertising for month-long campaign	332,000/y	Estimated from a similar statewide mass media campaign in North Carolina promoting seat belt use, in which advertising purchases were $52,907 per week for newspaper advertisements in 15 daily newspapers (1 advertisement per week for 4 weeks), 1,406 television spots, and 3,154 radio spots^b^ 1996 costs were adjusted for inflation to 2014 using the Consumer Price Index.
Content development (one-time)	368,000	From campaign promoting seat belt use described above^c^
**Free colonoscopy voucher for uninsured**		
Voucher for colonoscopy	750/person	2013 Medicare physician fee schedule (www.cms.gov) for Healthcare Common Procedure Coding System codes 45378, 45380, 45383, 45384, 45385, G0121

a Data sources: Lee et al (14) and Lewis et al (15).

b Data source: Broadwater et al (17).

c Data source: Williams et al (16).

The intervention consisting of mailing of reminders to the Medicaid registry involved mailing initial letters to all Medicaid enrollees turning 50 that provided information on the importance of CRC screening, recommended screening guidelines, information on available screening options, and instructions for scheduling a screening test or requesting additional information. A similar letter was sent each year for the 10-year study period to all Medicaid enrollees who were not up to date with CRC screening according to claims data, excepting people who were diagnosed with CRC. Reflecting data limitations, 10% of those who underwent CRC screening were assumed to have been erroneously sent a reminder because of imperfect data or data retrieval. Based on evidence from prior trials, being sent a reminder leads to a 5–percentage-point increase in the probability of being screened, recognizing that the effect of the mailing is greatest among those who receive and read the mailing and that there are many failure points for this intervention between being sent a mailing and receiving screening (13–15).

The endoscopy expansion intervention involved providing financial incentives for 6 new endoscopy facilities to be located in zip codes of greatest need, defined as the areas with the greatest density of unscreened adults residing more than 25 miles from a facility who could more readily reach a facility if it were located within 25 miles or less. The effect on screening resulted from the increased chance of screening associated with a decreased distance to a screening facility and increased number of screenings in the person’s county of residence in the statistical models — though these estimated effects were small and varied by insurance type (6).

The mass media intervention, which was targeted to African Americans, involved an annual month-long mass media campaign using tailored television, print, and radio advertisements to communicate the importance of CRC screening. On the basis of evidence, the campaign was assumed to reach 80% of African Americans state-wide, resulting in a 2–percentage-point increase in screening probability among those who were reached by the advertisements in that year (16–23). A residual effect was assumed to increase the probability of screening among 40% of non-African Americans by 1 percentage point (24). The persuasive impact of advertising decays quickly, so no lasting impact was assumed (25).

The fourth intervention simulated a statewide program in which uninsured people turning 50 were provided a voucher for a free colonoscopy until a maximum of 50 vouchers were used in each of the 10 intervention years. The vouchers provided payment directly to the endoscopy facility and included the initial cost of the colonoscopy and any polyp removal and biopsy.

## Results

Under the calibrated screening-as-usual scenario, 53.0% in 2014 and 54.0% in 2023 of age-eligible people were estimated to be up-to-date with CRC testing. Medicaid mailed reminders, mass media, and vouchers for the uninsured all produced modest increases in up-to-datedness overall (Figure and Table 3). When comparing the 4 interventions’ effects, the mailed reminder and mass media campaign produced the largest increases in CRC screening. The voucher program and endoscopy expansion had smaller, but positive, effects. Costs per additional life-year up to date ranged from less than $15 (mailed reminder) to more than $200 (expansion of endoscopy facilities). The combination of the mailed reminder and mass media interventions was most cost-effective, assuming a state’s willingness to pay approximately $20 per additional life-year up to date and invest $5.3 million overall.

**Figure:**
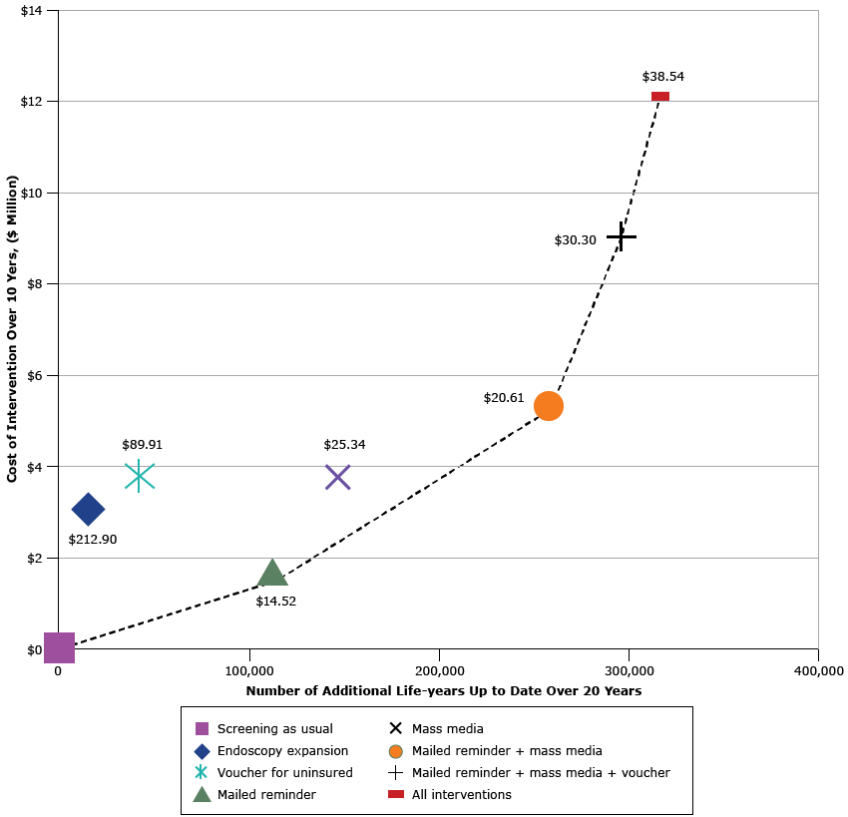
Cost-effectiveness efficiency frontier presenting the additional life-years up to date with recommended colorectal cancer screening among the population age-eligible for screening during the intervention window (*x*-axis) and intervention cost (*y*-axis) under each of 4 intervention scenarios, compared with screening as usual (existing screening). Results are presented for each intervention alone and for combination scenarios in which interventions are added, one at a time, based on their estimated intervention cost per additional life-year up to date (in 2014 US dollars). The dashed line indicates single and combined intervention scenarios that are not dominated by other intervention scenarios, where “dominated” means that the scenario is both more expensive and has less impact. Dollar values inside the figure indicate cost of each additional life-year up to date. **Table:** 

Intervention	Intervention Cost	Life-Years Up To Date	Cost of Each Additional Life-Year Up to Date, $
Screening as usual	0	0	0
Endoscopy expansion	3,000,000.00	14,094	212.9
Voucher for uninsured	3,750,000.00	41,709	89.9
Mailed reminder	1,619,578.00	111,516	14.5
Mass media	3,694,800.00	145,821	25.3
Mailed reminder + mass media	5,302,918.00	257,306	20.6
Mailed reminder + mass media + voucher	9,049,371.00	298,692	30.3
All interventions	12,049,517.00	312,648	38.5

**Table 3:** Simulated Age-Eligible^a^ North Carolina Population Up to Date With Recommended Colorectal Cancer Screening on December 31, 2023

Variable	Screening as Usual^b^, %	Percentage-Point Change in Testing Under Each Intervention Scenario Compared With Screening as Usual			
		Mailed Reminder	Endoscopy Expansion	Mass Media	Voucher for Uninsured
**Overall**	54.0	+0.4	+0.0	+0.5	+0.2
**By sex**					
Male	55.5	+0.3	+0.0	+0.6	+0.2
Female	53.0	+0.5	+0.0	+0.5	+0.1
**By race**					
White	55.4	+0.3	+0.0	+0.3	+0.1
African American	51.9	+0.9	+0.1	+1.4	+0.2
Other^c^	48.1	+0.5	+0.0	+0.4	+0.4
**By insurance**					
Private	57.0	+0.0	+0.0	+0.5	+0.0
Medicaid	50.3	+4.6	+0.2	+0.8	+0.0
Medicare	52.0	+0.0	+0.0	+0.4	+0.0
Medicare and Medicaid	44.8	+3.5	+0.1	+0.7	+0.0
Uninsured	14.6	+0.0	+0.0	+0.6	+1.2
**By county**					
Gap (maximum–minimum)^d^	15.7	−0.1	+0.0	+0.2	+0.0

a Aged 50 to 75 years.

b Existing screening practices.

c No distinctions by ethnicity (ie, Hispanic) possible in claims data.

d The disparity in percentage of population up to date with colorectal cancer screening between counties with the highest (maximum) and lowest (minimum) performance under screening as usual and the percentage point change in that gap under each intervention scenario.

Table 3 presents the impact of each intervention, independently, on the percentage of people up to date with CRC screening in 2023 among all subpopulations studied. Mailed reminders reduced the screening gap between Medicaid enrollees and the privately insured from 6.7 to 2.1 percentage points. The mass media intervention reduced the gap between whites and African Americans from 3.5 to 2.4 percentage points. The voucher for uninsured reduced the gap between privately insured and uninsured residents from 42.4 to 41.3 percentage points. Expansion of endoscopy facilities did not increase overall screening or reduce the gap between counties with the highest and lowest screening rates.

## Discussion

Analysis of the individual-based simulation model identified mailed reminders for Medicaid enrollees and a mass media campaign targeting African Americans to be the most effective and cost-effective options to increase CRC screening, assuming society is willing to pay approximately $25 per additional life-year a person is up to date with recommended screening. The proposed voucher program for the uninsured should be adopted only if addressing disparities in screening between uninsured and insured individuals is particularly important or if willingness to pay approaches $100 per additional life-year up to date with screening. Endoscopy expansion, as conceptualized, was relatively ineffective and strongly surpassed by the other interventions. Although some interventions were cost-effective, the interventions examined required 10-year investments ranging from $1.6 million to $3.75 million but had small effects individually, and in combination, on the state’s overall screening rates.

This study focused on the cost per additional life-year a person was up to date with CRC screening recommendations. This metric has the advantage of better weighting differential intervention effects on receipt of FOBT and colonoscopy, given the various recommended frequencies of these tests. Little guidance existed previously on what constitutes a reasonable cost per additional life-year up to date with CRC screening. To provide some perspective, a cost of $25 per life-year up to date would be equivalent to increasing the cost of annual FOBT from its current cost of approximately $20 to $45 each year, or the cost of one colonoscopy (which covers a person for 10 years) from its current cost of approximately $750 to $1,000. Previous studies have indicated that costs of these screening test would be within the range considered acceptable for such interventions (26).

Unlike most CRC models, the comparator scenario used in this study was based on state-specific data and explanatory statistical models, providing a current understanding of who is and who is not being screened and with which screening method in a full age-eligible population, and what might be gained through alternative interventions. This understanding can be used to inform intervention program planning aimed at improving CRC screening rates within resource constraints while simultaneously addressing disparities, as illustrated here. Although other methodological approaches, such as longitudinal, multiple case studies (27,28) and pragmatic trials (29) can also be used to gain valuable insights about what works where and why, such studies are expensive and take time. Because models like ours can compare multiple intervention programs, such approaches can guide efforts toward more efficiently closing gaps in CRC screening in a timely manner and can support prioritizing state-level planning and research funding, helping health officials and policy makers set more achievable goals (30).

This model has some limitations. First, not all factors that affect CRC screening (eg, changes in reimbursement policy or technology) could be modeled. Second, as with any simulation model, there is uncertainty about key model parameters, such as intervention cost and effectiveness. Additionally, the lack of a full cost accounting study limits the ability to reflect on long-term costs or savings that may result from these interventions. Specifically, this analysis does not account for the costs of cancer treatment (those costs resulting from detection and also those costs prevented), so the full economic impact or effects on illness and death are not reflected. Finally, the ability to measure the outcome is affected by both inaccuracies in claims-based and self-reported data, which we attempted to address by proportionally increasing all claims data-based screening probabilities to match estimates based on self-report (adjusted for self-report bias as described in the Methods section). This important limitation of existing data needs to be considered in establishing and evaluating national targets, including Healthy People 2020 targets, and in evaluating our ability to reach them.

Despite these limitations, a model such as this provides a useful foundation for informing intervention approaches, and it can be updated to support further integration of new data on costs, screening assumptions, and emerging evidence on best practices, with re-analysis informing ongoing intervention decisions. For example, decision makers might be interested in revising the mailed reminder if it was thought to be less expensive to integrate material into ongoing broad mailings at the state level (eg, to drivers for license and registration renewal) or more effective to include a fecal immunochemical test in the mailing. Alternatively, as evidence mounts about the feasibility of mobile endoscopy units, an intervention based on these units might be favored to enhance access. After updating intervention scenarios, analyses such as those presented here could be replicated or extended to update policy recommendations or address new population health, clinical, or service delivery questions. Across settings, various intervention scenarios can be developed to reflect priorities and preferences for interventions. Over time, as progress is made toward addressing disparities and closing gaps in screening, updating and re-analyzing the model could chart a dynamic course toward efficiently meeting established population health targets. Along the way, as outside factors change (eg, insurance expansion, changing reimbursement policies, new clinical guidelines), data should be re-analyzed to determine whether intervention programs should be altered. Also, analysis of a model such as ours could support logistical planning in North Carolina and elsewhere – for example, by examining whether existing endoscopy capacity is sufficient in light of Medicaid eligibility expansion.

Using simulation models to compare hypothetical programs to increase CRC screening is an important but underused strategy when planning programs to reduce the burden of CRC and related disparities in receipt of screening. Using state-specific and published data, the scientific literature, and decision-maker input to formulate intervention scenarios, our analysis of an individual-based simulation model suggested that mailed reminders to Medicaid beneficiaries and a mass media campaign targeted to African Americans would be among the most cost-effective strategies available to both increase the proportion of persons up to date with CRC testing and to decrease disparities in CRC screening by income, insurance status, and race in North Carolina with moderate investment. However, to ensure that greater proportions of the population are screened (eg, 70%–80%), substantially higher investment would be required in these or other interventions.
